# Blended sea level anomaly fields with enhanced coastal coverage along the U.S. West Coast

**DOI:** 10.1038/sdata.2016.13

**Published:** 2016-03-01

**Authors:** C.M. Risien, P.T. Strub

**Affiliations:** 1 College of Earth, Ocean and Atmospheric Sciences, Oregon State University, 104 CEOAS Administration Building, Corvallis, Oregon 97331-5503, USA

**Keywords:** Physical oceanography, Climate sciences

## Abstract

We form a new ‘blended’ data set of sea level anomaly (SLA) fields by combining gridded daily fields derived from altimeter data with coastal tide gauge data. Within approximately 55–70 km of the coast, the altimeter data are discarded and replaced by a linear interpolation between the tide gauge and remaining offshore altimeter data. To create a common reference height for altimeter and tide gauge data, a 20-year mean is subtracted from each time series (from each tide gauge and altimeter grid point) before combining the data sets to form a blended mean sea level anomaly (SLA) data set. Daily mean fields are produced for the 22-year period 1 January 1993–31 December 2014. The primary validation compares geostrophic velocities calculated from the height fields and velocities measured at four moorings covering the north-south range of the new data set. The blended data set improves the alongshore (meridional) component of the currents, indicating an improvement in the cross-shelf gradient of the mean SLA data set.

## Background & Summary

More than 20 years of altimeter data have greatly improved our understanding of upper ocean processes, including large scale ocean circulation^1,2^, mesoscale variability^3,4^, sea floor topography^5,6^, climate variability^7–9^, and the distribution within eddies of chlorophyll concentration^10^ and macrofuna feeding^11^. In coastal regions, however, altimeter observations are often of questionable accuracy due to factors including land contamination^12,13^, imprecise tidal corrections^14^ and incorrect removal of atmospheric effects^15,16^. These issues limit the use of altimeter-derived data products in coastal areas^17^, as discussed in detail in Vignudelli *et al.*^18^ and Cipollini *et al.*^19^. They further complicate the already difficult task of producing uniformly gridded fields from the sparsely sampled along-track data in coastal regions where space and time scales are shorter than in the open ocean. Specific efforts to correct and improve nearshore along-track altimeter data include the use of customized tidal modelling^20,21^, use of special editing and higher-rate data^22,23^, recomputing the atmospheric corrections^15,16,24^, and waveform retracking^13,25,26^.

Saraceno *et al.*^27^ were able to improve weekly mean, coastal sea level observations along the U.S. West Coast between 40° and 45°N for the 13-year period 1993–2005 by first removing all altimeter observations within 37 km of the coast. Based on data from five tide gauge stations located within the study area, Saraceno *et al.*^27^ then created a virtual array of low-pass filtered tide gauge stations at 0.2° intervals along the coast. Finally, the tide gauge derived sea level data were interpolated from the coast to the offshore AVISO fields using the Delaunay triangulation method^28,29^. Compared to the original AVISO data, this methodology significantly improved the accuracy of alongshore geostrophic currents derived from their blended, weekly SLA data set, in comparison to in situ current meter observations over the shelf.

The data set presented here extends the work of Saraceno *et al.*^27^ by improving the temporal resolution from weekly to daily SLA fields, over a longer period, and by expanding the region to include the entire U.S. West Coast. We use an inverse-distance weighted interpolation method to blend low-pass filtered, daily mean tide gauge observations with daily, 0.25° latitude x 0.25° longitude AVISO SLA fields. This data set covers the 22-year period 1 January 1993–31 December 2014 from 32–48.5°N and 135–115°W. It should be emphasized that the data set we are producing does not attempt to retrieve improved altimeter data in the coastal domain. As in Saraceno *et al.*^27^, it substitutes tide gauge data in the region within approximately 55–70 km of the coast for the problematic altimeter data to produce a blended SLA data set.

Much of our validation (see the ‘Technical Validation’ section below) of the improvement of the new data set, in comparison to the original AVISO daily data, also follows Saraceno *et al.*^27^ by comparing geostrophic velocities derived from the SLA fields to observed velocities from moorings, now stretching from northern Washington to southern California. This comparison amounts to an indirect validation of the gradients in the surface height fields and requires some discussion. Even if the altimeter fields perfectly represent the ocean surface dynamic height fields, there will be differences between geostrophic currents derived from the gradients of those heights (as calculated over some finite distance) and velocities measured within the water column by current meters at single locations. These differences are discussed further in the ‘Technical Validation’ section. We emphasize, however, that the primary data set produced here consists of SLA height fields. For convenience, we also provide geostrophic velocity fields calculated in the simplest manner (centered differences over approximately 40–50 km). Those preferring to use more sophisticated methods of calculating gradients should use the SLA fields to do so.

The paper is organized as follows: in the Methods and Data Record sections we describe the data sets and methods used to generate the blended AVISO-tide gauge data set (referred to as AVISO+TG hereafter) as well as the in situ velocity observations used to validate and verify this data set. The results of our validation and verification efforts are described in the Technical Validation section.

## Methods

### AVISO altimeter fields

The 0.25° latitude × 0.25° longitude gridded SLA altimeter fields were produced by DUACS/SSALTO (Data Unification and Altimeter Combination System/Segment Sol multi-missions d’ALTimetrie, d’orbitographie et de Localisation précise) and distributed by CLS (Collecte Localis Satellites) and AVISO (Archivage, Validation, Interprétation des données des Satellite Océanographiques). The data are available at http://www.aviso.altimetry.fr/en/data.html. Here we use the DUACS 2014 (v15.0) Delayed Time (DT) ‘all-sat-merged’ daily mean fields^30^ for the period 1 January 1993 through 31 December 2014. The ‘all-sat-merged’ fields consist of datasets from up to four satellites at any given time. Using all available missions for a given time period improves sampling and long wavelength error determination, thus producing a higher quality data set, but one that is not homogeneous over the entire time span of the data record. DUACS uses a 20-year reference period of 1993–2012 to adjust each of the along-track data sets to match the ‘reference’ altimeters (TOPEX/Poseidon, Jason-1 and Jason-2), which span the entire time period. They then apply an offset to each along-track data set to make it consistent with a global mean SLA value of zero during 1993, before processing and mapping the along-track data onto their global grid. Thereafter, finding the global mean value from the gridded data during any subsequent period shows the mean global sea level rise since 1993.

### Tide gauge observations

**Figure 1:**
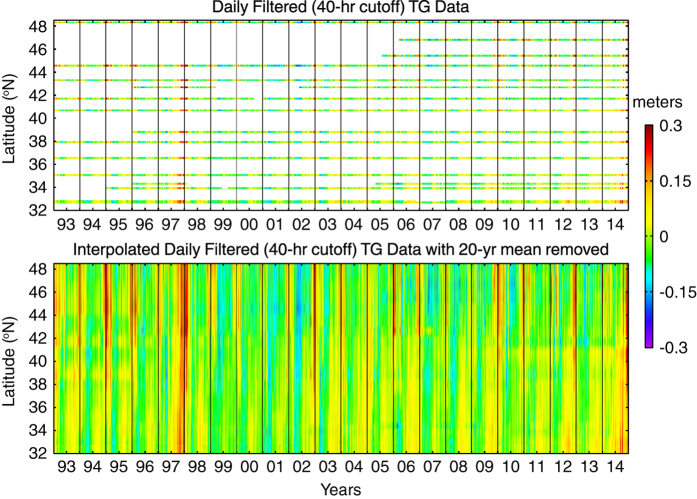
Time series of 40-hour low-pass filtered, daily averaged SLA as measured by the sixteen US. West Coast tide gauge stations (top). Note there are two nearby tide gauges at the southern location (see Fig. 2). Results of the interpolation of the sixteen tide gauge station time series to a high resolution alongshore grid (bottom). To be consistent with the AVISO fields, a 20-year mean (1993–2012) was subtracted from each of the interpolated tide gauge time series. The thin, black vertical lines indicate January 1 for each of the 22 years.

**Figure 2:**
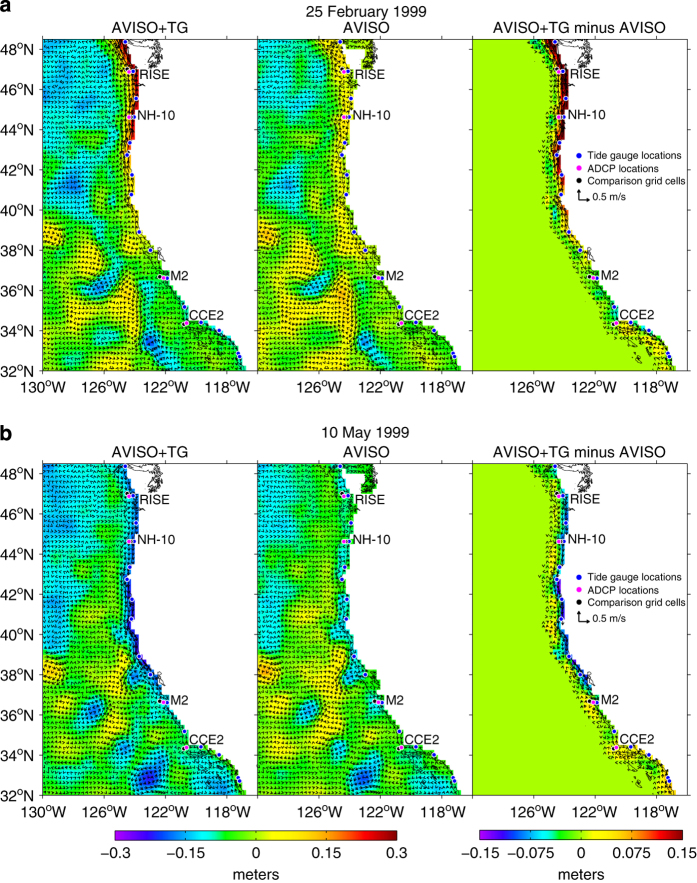
The left panels show the AVISO+TG daily SLA and geostrophic surface velocity (vectors) fields for 25 February 1999 (**a**) and 10 May 1999 (**b**). The middle panels show the original daily AVISO fields for 25 February 1999 (**a**) and 10 May 1999 (**b**). The right panels show the difference (AVISO+TG minus AVISO) between the AVISO+TG fields and the adjusted AVISO fields. Also shown are the locations of the tide gauge stations (blue dots), the 4 mooring locations (magenta dots), and the grid cell locations (black dots) that were compared to the ADCP data. The black line indicates the 200 meter isobath.

The National Oceanic and Atmospheric Administration’s (NOAA) Center for Operational Oceanographic Products and Services (CO-OPS) manages the National Water Level Observation Network (NWLON), a network of long-term, continuously operating water level stations throughout the United States and its territories. Verified, hourly mean sea level data for 16 U.S. West Coast tide gauge stations (Station ID 9443090; 9441102; 9437540; 9435380; 9432780; 9431647; 9419750; 9418767; 9416841; 9415020; 9413450; 9412110; 9411340; 9410840; 9410230; 9410170) for the period of 1993–2014 were obtained from NOAA CO-OPS (http://tidesandcurrents.noaa.gov). The time series and locations of each of these stations are shown in Fig. 1 and by the blue dots in Fig. 2. The Mean Sea Level tidal datum was used in this analysis.

### NCEP reanalysis I fields

The atmospheric surface pressure fields used in the inverse barometer ‘correction’ to tide gauge heights come from the National Centers for Environmental Prediction (NCEP) Reanalysis I project, using an analysis/forecast system to perform assimilation of past data from 1948 to the present^31^ (data available at http://www.esrl.noaa.gov/psd/data/gridded/data.ncep.reanalysis.surface.html). The dynamical model and data assimilation system remains unchanged over the reanalysis period. While this avoids perceived climate jumps associated with changes in the operational data assimilation system, the reanalysis system is still affected by changes in assimilated observations^32^. For the work presented here, six-hourly surface pressure fields for the period 1 January 1993–31 December 2014 were linearly interpolated so as to correspond to the hourly tide gauge observations described above.

Ablain *et al.*^9^ report significant improvements in sea level height estimates at mesoscale and regional spatial scales using ECMWF (European Centre for Medium-Range Weather Forecasts) ERA-Interim Reanalysis^33^ fields rather than operational ECMWF fields to calculate certain atmospheric corrections. In an effort to validate our decision to use the NCEP Reanalysis fields here rather than, for example, the ERA-Interim fields we calculated the root mean square (RMS) error between daily mean sea level pressure data collected by NOAA Buoy 46050 (http://www.ndbc.noaa.gov/station_page.php?station=46050) and the ERA-Interim and NCEP Reanalysis grid cells closest to the 46050 location (44.656°N, 124.526°W) for the period 1993–2014. We find the RMS errors for the ERA-Interim and NCEP Reanalysis fields to be 1.067 and 1.014 hPa, respectively. Given that atmospheric pressure is the only variable used from the atmospheric models and the RMS error is smaller for the NCEP Reanalysis fields, we consider them to be of sufficient quality to be used in the generation of our blended AVISO+TG data set.

### *In situ* current velocities

In this study, we use in situ time series of current velocities estimated from Acoustic Doppler Current Profilers (ADCP) that were mounted on four moorings located off the coasts of Washington, Oregon and California (Supplementary Table 1). An indirect validation of the altimeter SLA fields is carried out through comparisons between these in situ water velocities and geostrophic velocities derived from the AVISO+TG and AVISO data sets. The locations of these four moorings are shown in Fig. 2 (magenta dots).

The RISE (River Influences on Shelf Ecosystems) NOrth (RINO; data available at http://www.bco-dmo.org/dataset/3586/data) mooring was deployed off the coast of Washington as part of a multi-year, National Science Foundation funded, interdisciplinary study of the Columbia River plume^34^. The mooring was deployed in 2005 and 2006 for the months of mid-May through September on the continental shelf at the 80 meter isobath at 47.01°N, 124.49°W, approximately 25 km west of Grays Harbor, WA.

The long-term mooring site on the Oregon shelf known as NH-10 was established in close proximity to the Newport Hydrographic (NH) line^35^ in August 1997 (ref. 36) as part of the U.S. GLOBal Ocean ECosystems Dynamics (GLOBEC) program. The NH-10 mooring, which was funded by the GLOBEC program through 2004, is located at approximately 44.64°N, 124.3°W, on the continental shelf at the 81 meter isobath almost 20 km due west of Newport, OR. Since 2006 the mooring has been maintained and operated by Oregon State University (OSU) with funding from the Northwest Association of Networked Ocean Observing Systems (NANOOS) and the Center for Coastal Margin Observation & Prediction (CMOP).

The Monterey Bay Aquarium Research Institute (MBARI) M2 mooring (data available at http://dods.mbari.org/lasOASIS/) was deployed approximately 50 km west of Monterey Bay at 36.70°N, 122.39°W. While this mooring, which is located on the continental slope in about 1,800 meters of water, was maintained for the 19-year period 1992–2010, ADCP data are only available for the years 1998–2009 with a significant data gap extending from 2001–2003.

The California Current Ecosystem coastal upwelling mooring (CCE-2; data available at http://mooring.ucsd.edu/index.html?/projects/cce/cce2_data.html) is operated as a collaboration between Scripps Institution of Oceanography, the NOAA PMEL (Pacific Marine Environmental Laboratory) carbon and ocean acidification group, the NOAA Southwest Fisheries Science Center, and University of California, Santa Barbara. The mooring, which was first deployed in 2010, is located at 34.2°N, 120.7°W, approximately 35 km west of Point Conception on the continental slope in approximately 770 meters of water.

The ADCP bin depths 16.5, 16.5, 15 and 17 meters for each of the four moorings RINO, NH-10, M2 and CCE-2, respectively, were chosen based on data availability as well as an attempt to select a bin depth that was common and consistent across all four moorings, while also being below most of the effects of wind-driven Ekman currents. All ADCP data were hourly averaged and low-pass filtered using the same 40-hour Loess filter^37^ as was used for the tide gauge data. The low-pass filtered data were then averaged to create daily mean time series. Finally, the mean for each mooring time series was removed. When forming differences between a time series from a mooring and altimeter-derived velocities, the mean of the altimeter-derived data set was removed over a period identical to the mooring period.

### Synthetic tide gauge stations

An inverse barometer (IB) correction was applied to each of the 16 tide gauge station hourly time series according to the following equation^38^:

(1)IB=−9.948*(Patm−1013.3),

where P_atm_ is the time varying atmospheric pressure derived from six-hour NCEP Reanalysis I sea level pressure fields. The scale factor 9.948 is based on the empirical value of the IB response at mid-latitudes^39^. The hourly IB corrected tide gauge data were then low-pass filtered using the same 40-hour Loess filter^37^ used for the ADCP velocity data and averaged to create daily mean time series. Given the removal of high frequency signals by the low-pass filter and daily averages, a Dynamical Atmospheric Correction was not used.

An along-coast 0.125° grid was created between 32 and 48.5°N. Each of the 16 tide gauge time series was assigned the latitude of the nearest of these grid cells (Fig. 1, top panel). The data were then spatially interpolated at each time step using an inverse-distance weighted interpolation method^40^ to create a synthetic, gap-free tide gauge data set that consisted of 133 time series. To maintain consistency with the 20-year reference period of the AVISO fields, a 20-year mean (1993–2012) was subtracted from each of the 133 time series to produce the final field of synthetic coastal tide gauge sea level anomalies (Fig. 1, bottom panel). Finally, the 0.125° synthetic tide gauge data set was averaged to the 0.25° AVISO latitude grid and mapped to the most coastal grid cell of the AVISO grid next to the U.S. West Coast between 32 and 48.5°N.

### Blending AVISO and tide gauge observations

Daily, gridded SLA fields for the period 1 January 1993–31 December 2014 and region 32–48.5°N and 135–115°W were extracted from the global AVISO fields. Because the mean AVISO SLA gridded values over the 20-year reference period from 1993–2012 are not zero, the 20-year mean was formed and removed at each grid point, for consistency with the tide gauge data. This mean is the average sea level rise during the 20-year reference period, approximately 2.5 cm. It also includes spatial variability in the form of noise, with magnitudes of several millimetres. This is created by imprecisions in the mean sea surface and other details of the processing used to map the data on to a uniform grid by DUACS. After removing the mean and noise, we refer to this data set as the ‘adjusted’ AVISO SLA fields. Next, all AVISO observations within 3 grid cells (approximately 55–70 km) of the coast were removed (at the two most northern lines of grid points, it was necessary to remove 5 and 6 grid points, respectively, to eliminate noise caused by stronger tides and a relatively wide shelf near the mouth of the Juan de Fuca Straits). Using the inverse-distance weighted interpolation method referenced above^40^, we interpolated between the tide gauge data at the coast and the remaining daily offshore adjusted AVISO data to generate the final, blended AVISO+TG data set. If one wants to ‘re-adjust’ these fields to match the original AVISO fields, the mean of the original fields at each grid point over the 20-year reference period should be formed and added to our AVISO+TG data set. For convenience, this 20-year (1993–2012) mean SLA field is provided with our AVISO+TG SLA fields.

### Geostrophic current estimates

Following methods described in Saraceno *et al.*^27^, geostrophic currents were estimated for each of the daily AVISO+TG fields. The zonal and meridional geostrophic velocity components at each grid point were estimated using centered differences as:

(2)u(x,y)=−(gf)⋅SSHx,y+1−SSHx,y−1d(x;y+1,y−1)

(3)v(x,y)=(gf)⋅SSHx+1,y−SSHx−1,yd(x+1,x−1;y),

respectively, where *f* is the Coriolis parameter, *g* is the gravitational acceleration and *d* is the distance between the grid points used in the calculation (approximately 40–50 km). To estimate values as close to the coast as possible, SLA data adjacent to the coast were linearly extrapolated to values at the next gridpoint (over the land) before using the centered difference formula at the grid point next to the coast. The same equations were used to derive geostrophic velocities for each of the original daily mean AVISO fields and our adjusted AVISO fields. Given the smoothing inherent in the creation of the gridded SLA fields, the gradients calculated over these approximately 40–50 km distances proved suitable for the validation efforts and provided velocities on as small a scale as possible for comparison to the current meters.

## Data Records

We form a new data set of blended SLA fields by combining gridded daily fields derived from altimeter data with coastal tide gauge data. Within approximately 55–70 km of the coast, the altimeter data are discarded and replaced by a linear interpolation between the tide gauge and remaining offshore altimeter data. A 20-year mean is subtracted from each time series (tide gauge or altimeter) before combining the data sets to form the blended sea level anomaly data set. Geostrophic velocity anomaly fields are formed from the surface heights.

For each year (1993–2014) daily mean SLA, as well as u and v geostrophic velocity anomaly fields are made available as CF compliant NetCDF files. The 22 individual NetCDF files have been added to a single TAR (Tape ARchive) file, which can be accessed at https://ir.library.oregonstate.edu/xmlui/handle/1957/57170 (Data Citation 1). Each NetCDF file contains the latitude and longitude grid cell coordinates where the values provided indicate the center of each grid cell. These grid cell coordinates are identical to the original 0.25° AVISO fields. The gridded 20-year (1993–2012) mean of the SLA fields that was removed is also made available, for those wishing to recreate the original AVISO fields in the offshore region.

## Technical Validation

### Synoptic scale variability

Examples of the blended AVISO+TG daily SLA and derived geostrophic velocity fields are shown in Fig. 2a,b (left panels) for downwelling and upwelling periods, respectively. These are typical of winter and spring conditions in the northern California Current System (CCS). The original AVISO daily mean fields are shown in the middle panels. The differences between our AVISO+TG data and the adjusted AVISO data are presented in the right panels, where only non-zero values are represented by the vectors and colours next to the coast in the region where AVISO data were removed and replaced by the interpolated tide gauge data.

During periods of downwelling-favorable winds, such as occurred on 25 February 1999 (Fig. 2a), the AVISO+TG fields tend to have a stronger and more continuous positive SLA signal along the coast (more than 10 cm higher) between 38° and 48.5°N, relative to AVISO fields. During periods of upwelling-favorable winds, such as occurred on 10 May 1999 (Fig. 2b), the pattern is reversed with relatively low SLA occurring along the coast between 34° and 48.5°N. The relatively high (low) SLA along the coast results in northward (southward) alongshore current velocities over the shelf that are several 10’s of cm s^−1^ greater and often opposite in direction to those derived from AVISO fields alone.

**Figure 3:**
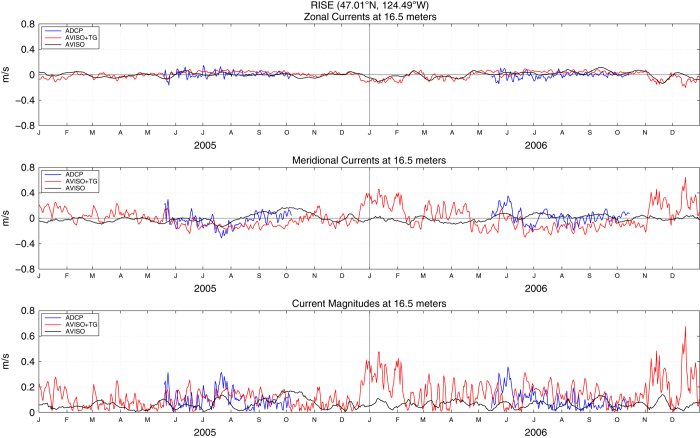
Zonal (top) and meridional (middle) geostrophic velocities as well as geostrophic current magnitudes (bottom) estimated from AVISO (black lines) and AVISO+TG (red lines) fields at 46.875°N, 124.375°W. Also shown are zonal (top) and meridional (middle) current velocities as well as current magnitudes (bottom) as measured by the RISE ADCP (47.01°N, 124.49°W) at 16.5 m depth (blue lines). The mean value for each zonal and meridional time series, for the period presented here, was removed.

**Figure 4:**
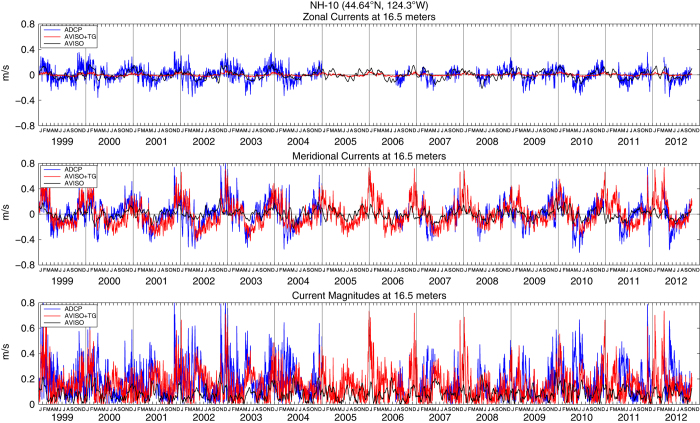
Zonal (top) and meridional (middle) geostrophic velocities as well as geostrophic current magnitudes (bottom) estimated from AVISO (black lines) and AVISO+TG (red lines) fields at 44.625°N, 124.375°W. Also shown are zonal (top) and meridional (middle) current velocities as well as current magnitudes (bottom) as measured by the NH-10 ADCP (44.64°N, 124.3°W) at 16.5 m depth (blue lines). The mean value for each zonal and meridional time series, for the period presented here, was removed.

**Figure 5:**
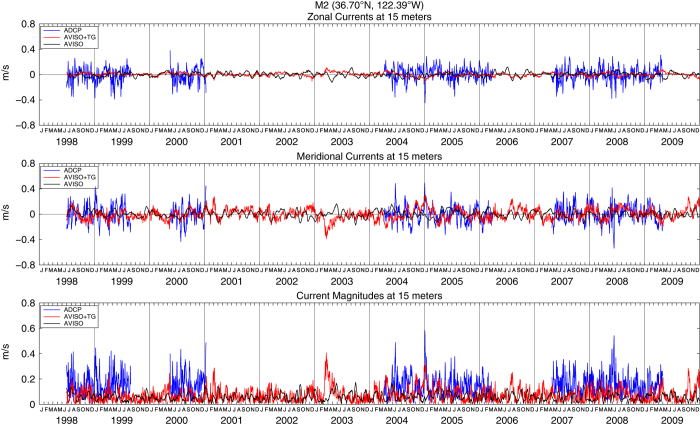
Zonal (top) and meridional (middle) geostrophic velocities as well as geostrophic current magnitudes (bottom) estimated from AVISO (black lines) and AVISO+TG (red lines) fields at 36.625°N, 122.125°W. Also shown are zonal (top) and meridional (middle) current velocities as well as current magnitudes (bottom) as measured by the MBARI M2 ADCP (36.70°N, 122.39°W) at 15 m depth (blue lines). The mean value for each zonal and meridional time series, for the period presented here, was removed.

**Figure 6:**
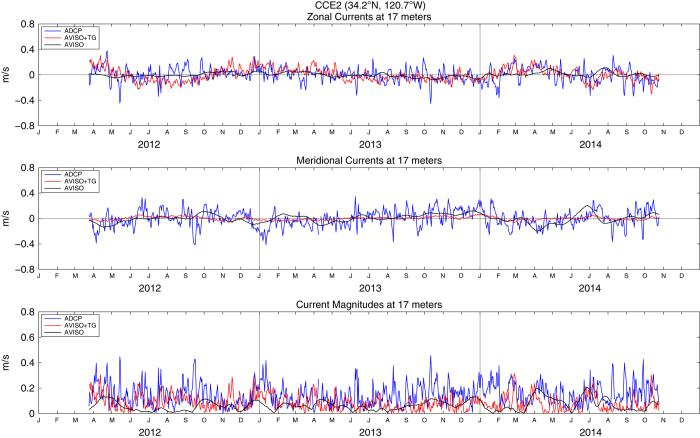
Zonal (top) and meridional (middle) geostrophic velocities as well as geostrophic current magnitudes (bottom) estimated from AVISO (black lines) and AVISO+TG (red lines) fields at 34.375°N, 120.625°W. Also shown are zonal (top) and meridional (middle) current velocities as well as current magnitudes (bottom) as measured by the CCE-2 ADCP (34.2°N, 120.7°W) at 17 m depth (blue lines). The mean value for each zonal and meridional time series, for the period presented here, was removed.

These enhanced coastal fields in the example daily AVISO+TG sea surface heights and velocities (Fig. 2a,b) respond rapidly to 2–8 day synoptic scale wind forcing^41^ associated with wintertime storms and summertime upwelling and relaxation events. The fact that most of the observed daily variability results from the tide gauge data is demonstrated by examination of the geostrophic velocities derived from the heights. These are compared to nearby in situ ADCP current measurements from the RINO, NH-10, M2 and CCE-2 moorings in Figs 3,4,5,6, respectively. For each comparison, the mean values for each time series for the common periods were removed. The locations of these four moorings as well as the comparison grid cells are shown as black and magenta circles in Fig. 2, respectively. Summary statistics (standard deviations) of zonal and meridional current velocities as well as current magnitudes for all four mooring locations are presented in Table 1. Summary statistics of the differences (ADCP minus AVISO[+TG]) as well as the correlation results are shown in Tables 2,3,4,5. All statistics are calculated for the times of available current meter data at each location.

Before examining the details of the figures and tables, several characteristics of the measured and geostrophic velocities should be noted. The comparisons are presented as an indirect validation of the SLA fields, since they can only validate the gradients in the height fields used in the geostrophic calculation, not absolute heights or offsets in those heights. On the current meter side, the mooring observations are of complete velocities, including ageostrophic components such as the wind-driven Ekman transports and smaller-scale motions. Previous studies of coastal upwelling systems have confirmed an approximate geostrophic balance for the alongshore component of observed velocities, which is not true for the cross-shore components. Since coastal winds are often polarized in the alongshore direction, cross-shore currents are strongly affected by onshore-offshore Ekman transports in the upper ocean and return flow below that. Off Oregon, early analyses of current meter velocities and hydrographic surveys by Smith^42^ found alongshore currents to be in approximate geostrophic balance, with horizontal scales between 50 and 70 km. In a coastal region off northern California, this was quantified using shipboard ADCP currents and CTD surveys by Kosro and Huyer^43^, finding a correlation of r=0.73 between the measured currents at 30 m and the cross-current pressure gradients. Their data also confirmed that currents over the shelf were more strongly polarized into the alongshore directions than farther offshore. Off central California in deep water approximately 500 km offshore, similar correlation values of 0.6–0.7 were found between current meter and geostrophic velocities calculated from altimeter data along tracks that crossed over the current meter location^44^. Thus, correlation values of approximately 0.6–0.7 serve as a benchmark for our correlations between observed and geostrophic currents. The altimeter height fields also contain noise, which is amplified by the spatial differences used in the geostrophic calculation, more so for differences calculated over short distances. The geostrophic velocities in the above studies used differences over scales of 40–60 km, similar to the approximately 40–50 km differences used here. Thus, our comparisons and correlations are expected to be similar to the above studies in the degree to which they are affected by amplified noise and ageostrophic motion in the moored data sets. They are also likely to be most useful when considering the alongshore currents, rather than the cross-shore currents. Staying within the AVISO grid, we calculate meridional and zonal components of the geostrophic and ADCP velocities, which are approximately in the alongshore and cross-shore directions, respectively, given the generally north-south direction of the coastline.

For completeness in Figs 3,4,5,6 we show the zonal and meridional components of velocity in the upper and middle panels, although we expect the meridional components to be more nearly alongshore and thus more closely in geostrophic balance. This is especially true at the two more northern locations, where the coastline is nearly north-south and the current meters are over the shelf, closer to the coast and more polarized in the alongshore direction. In the two southern locations, the coast is oriented more NW to SE, the current meters are farther offshore over the slope and the comparison geostrophic gridpoints are even farther offshore of the current meter.

Tables 2,3,4,5 show that correlations of the meridional components of the daily mean ADCP and altimeter-derived currents are higher for the AVISO+TG than the AVISO SLA for all except the most southern mooring, where they are the same. The average correlation of all four mooring locations increases from 0.37 to 0.49 for the AVISO+TG meridional currents. There are higher correlations for the two more northern moorings and the best comparison is found at NH-10, the location with the longest record and most energetic measured current magnitudes. Correlations at NH-10 between the ADCP and SLA-derived meridional currents increase from 0.51 for AVISO to 0.73 for AVISO+TG (Table 3). The value of 0.73 is similar to benchmark noted above, although it is slightly lower than the correlation of 0.83 at the same mooring that Saraceno *et al.*^27^ calculated using weekly mean observations and a shorter record. The standard deviation of the difference between moorings and altimeter-derived velocities also decreases for the AVISO+TG fields, again more notably at the NH-10 location.

To test whether correlations might be greater deeper in the water column, farther removed from Ekman layer effects, the ADCP meridional velocities at NH-10 from approximately 30 meters were also compared with the meridional geostrophic velocities derived from the AVISO+TG and AVISO data sets. Correlations between the ADCP and altimeter-derived meridional currents were 0.46 for AVISO and 0.68 for AVISO+TG, lower than the values observed at 16.5 meters. Thus we do not believe that the use of deeper observed currents would avoid Ekman effects and improve the comparisons.

Statistics are mixed for the zonal components of velocity, generally associated with the cross-shelf directions. Correlations between measured and geostrophic velocities are generally very low, except at NH-10. Although the correlations increase slightly from 0.55 to 0.59 for the AVISO+TG geostrophic velocities at that location, the standard deviation of the differences between measured and geostrophic velocities also increases for the AVISO+TG SLA. Thus, no clear picture emerges from comparisons of the zonal components, which are not expected to provide a good test of the SLA fields through geostrophic balances.

The velocity magnitudes are most useful in comparisons of their standard deviations, indicating the relative energy in the currents. At all four locations (Table 1), the AVISO+TG geostrophic currents are more energetic than the AVISO currents, and both are weaker than the measured currents. At all except the most southern locations, the increased energy is due to the meridional component, as evident in the Figs 3,4,5,6. Supplementary Fig. 1 presents an expanded view of the comparison at NH-10 for the years 2002 through 2004. Looking at the meridional component, periods of agreement and disagreement can be found, but it is clear that the tide gauge data have allowed the AVISO+TG SLA-derived velocities to respond to the synoptic forcing in a relatively realistic manner. The AVISO-only velocities, in contrast, are much less energetic, have almost no variability at time scales shorter than one month and often appear to be out of phase with the measured currents even on monthly time scales. A somewhat similar pattern is seen at Grays Harbor, where the record is short enough to allow examination of synoptic fluctuations. At the two southern locations, the zonal component becomes more energetic, to the extent that at CCE-2 the zonal component of the AVISO+TG velocities appear to contain most of the synoptic variability. Given the change in coastal orientation and the more offshore location, it is possible that the coastal jet at this more offshore site has been diverted partially into the zonal orientation. This is suggested by the fact that the correlations between ADCP and geostrophic currents in the zonal orientation at CCE-2 increases from 0.20 for AVISO to 0.29 for AVISO+TG currents. Overall, the velocities at the southern moorings that are over the continental slope and more distant from the coast provide less useful validations than the moorings over the shelf at the northern locations.

### Long-term and seasonal variability

**Figure 7:**
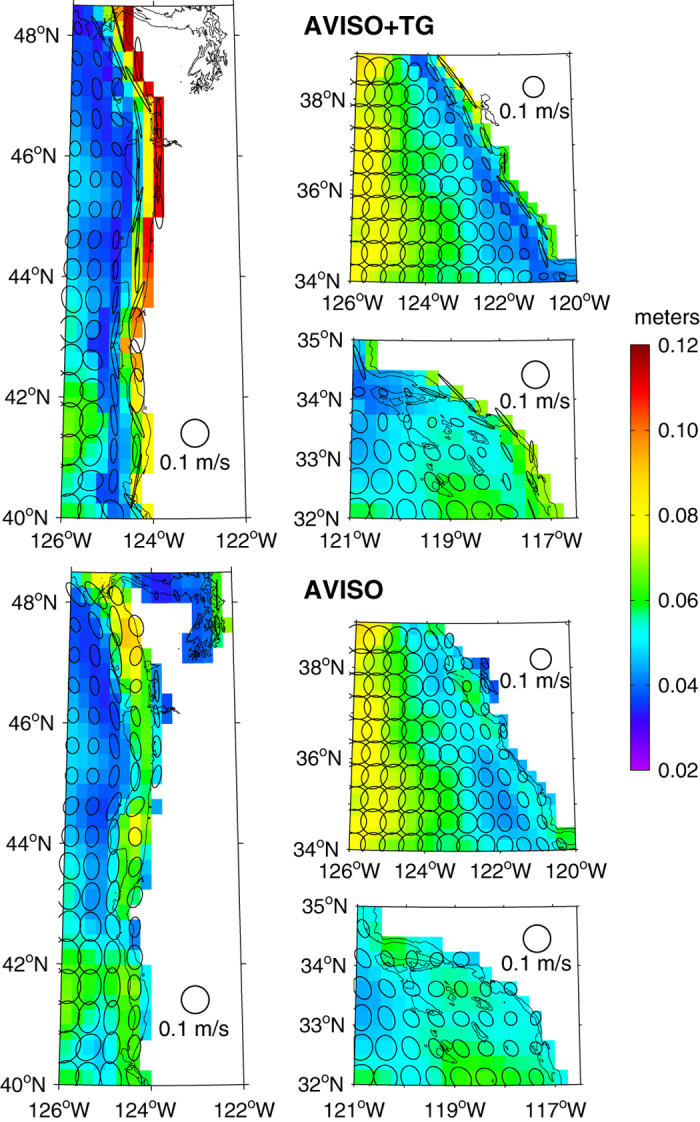
Standard deviation (1 January 1993–31 December 2014) fields are shown in colour for SLA for AVISO+TG (top) and AVISO (bottom). Overlaid are variance ellipses showing geostrophic current standard deviations as the lengths of the principal and secondary axes. Every second ellipse is plotted for clarity. The black line indicates the 200 m isobath.

The long-term (1 January 1993–31 December 2014) standard deviations of the AVISO+TG fields are shown in Fig. 7 (top). The velocity standard deviations represented by the principal axis ellipses and surface height standard deviations represented by the colours both indicate the relatively higher variability occurring along the coast between 32° and 48.5°N, in comparison to the AVISO fields (Fig. 7, bottom). The polarization of the variance ellipses in Fig. 7 confirms that the AVISO+TG geostrophic currents have greater alongshore variability relative to the AVISO currents, which is quantified by the standard deviation values presented in Table 1. They also demonstrate the rapid decrease in the polarization of the altimeter-derived velocities as one moves offshore, which affects the comparisons at the southern locations (36.7°N and 34.2°N).

The January AVISO+TG climatology field (Supplementary Fig. 2; top left panel) has elevated SLA near the coast north of 40°N relative to the January AVISO climatology field (bottom left). In comparison to the AVISO fields, these elevated SLA occur closer to the coast, i.e. to the east of the 200 m isobath (black line). South of 40°N and offshore, the AVISO SLA are slightly higher than the AVISO+TG field. In April, the AVISO+TG SLA are more negative next to most of the U.S. West Coast than the AVISO field. Likewise, SLA values are more negative north of 41°N in the July and October AVISO+TG fields, relative to AVISO. These comparisons demonstrate that the AVISO+TG fields have stronger signals next to the coast in monthly fields, as they do in the daily fields (Fig. 2). In the offshore region, the slightly higher SLA values of the AVISO fields reflects the fact that the long-term mean of the AVISO fields during the 20-year reference period is approximately 2.5 cm, rather than zero for the AVISO+TG fields.

### Interannual Variability

**Figure 8:**
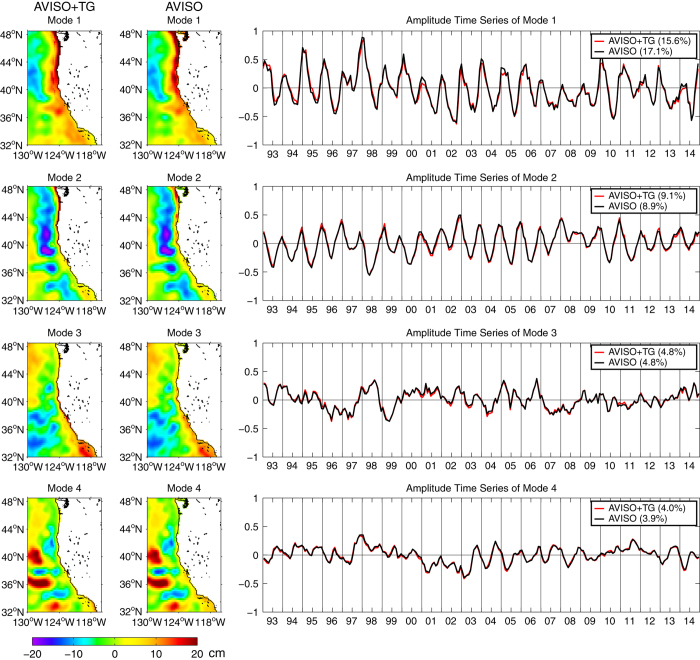
First four EOF modes of the AVISO+TG and AVISO fields. The amplitude time series of the AVISO+TG (red) and AVISO (black) fields are shown to the right.

Figure 8 shows that EOF analyses using monthly mean AVISO and AVISO+TG SLA fields yield very similar results for the first four modes of variability, which combine to explain approximately one third of the total variance. The first two modes are dominated by the seasonal cycle, with peaks and troughs in winter and summer, respectively. The first mode accounts for 17.1 and 15.6% of the variances, respectively, for AVISO and AVISO+TG. The wide band of high SLA next to the coast shows that this mode corresponds to the large-scale California Current north of 36°N, with poleward and equatorward flow anomalies in winter and summer, respectively. The second mode represents 8.9 and 9.1% of the variance in the AVISO and AVISO+TG fields, respectively, and is concentrated in a narrow band next to the coast over the shelf. The seasonal timing of the second mode SLA anomalies is the same as for the first mode. For both first and second modes, the spatial patterns of the AVISO+TG data produce stronger and more continuous signals next to the coast than the AVISO data. The third mode accounts for 4.8% of the variance for both the AVISO and AVISO+TG fields, with a spatial field that is broader south of 38°N and is especially strong in the Southern California Bight. This mode appears to be related in part to the El Niño Southern Oscillation (ENSO), with the amplitude time series showing prolonged positive values in 1997–1998 (a very strong El Niño) and positive peaks during 2002–2003 (a moderate El Niño), 2004–2005 and 2006–2007 (weak El Niños), where the strength of the El Niños are characterized by the Oceanic Niño Index (ONI) at the NOAA Climate Prediction Center, available at http://www.cpc.noaa.gov/products/precip/CWlink/MJO/enso.shtml. See also McClatchie *et al.*^45^ and Wolter and Timlin^46^. All four modes can contribute to interannual variability, as seen during the strong 1997–1998 event, while the moderate event in 2002–2003 receives contributions from the first three modes (especially mode 2). A moderate event in 2009–2010 is more strongly influenced by modes 1 and 2, as is a weak event in 1994–1995. The mode 3 amplitude time series contains negative values during La Niña events that are considered moderate (1998–1999, 2007–2008) and weak (1995–1996), based on the ONI index and references above. The similarity of the EOF modes (AVISO+TG versus AVISO) indicates that the use of the AVISO+TG data set causes no problem in analyses of interannual variability, as represented by the dominant EOF modes. It does, however, strengthen the interannual signals in the region adjacent to the coast.

It should be noted that the AVISO+TG mode 1 spatial pattern does show a discontinuity where the offshore AVISO fields intersect with the nearshore interpolated data. This can be noticeably reduced by applying a 3×3 grid cell median filter to the monthly SLA fields prior to the EOF calculation, without significantly impacting the structures visible in the AVISO+TG mode 1 spatial pattern. A 5×5 median filter removes the discontinuity completely but also reduces the extremes in the large-scale spatial patterns (Supplementary Fig. 3).

## Additional Information

Supplementary Information accompanies this paper at http://www.nature.com/sdata

**How to cite this article:** Risien, C. M. & Strub, P. T. Blended sea level anomaly fields with enhanced coastal coverage along the U.S. West Coast. *Sci. Data* 3:160013 doi: 10.1038/sdata.2016.13 (2016).

## Supplementary Material



Supplementary Information

## Figures and Tables

**Table 1:** Standard deviations of the current velocities and speeds estimated from AVISO SLA, AVISO+TG SLA fields and ADCP measurements.

	**s.d. meridional velocities (ms^−1^)**				**s.d. zonal velocities (ms^−1^)**				**s.d. velocity magnitudes (ms^−1^)**			
	**RISE**	**NH-10**	**M2**	**CCE-2**	**RISE**	**NH-10**	**M2**	**CCE-2**	**RISE**	**NH-10**	**M2**	**CCE-2**
AVISO	0.062	0.075	0.055	0.068	0.032	0.064	0.034	0.035	0.036	0.048	0.030	0.042
AVISO+TG	0.076	0.179	0.083	0.026	0.027	0.016	0.027	0.101	0.055	0.108	0.050	0.060
ADCP	0.112	0.202	0.135	0.131	0.046	0.105	0.108	0.112	0.070	0.132	0.083	0.085

**Table 2:** Standard deviations and correlations of the differences between current velocities and speeds estimated from AVISO SLA, AVISO+TG SLA fields and RISE ADCP (16.5 m depth) measurements as well as the slopes and y-intercepts of the linear regressions.

	**s.d. (ms^−1^)**	**R (ADCP versus AVISO(+TG))**	**Slope**	**Intercept**
U-ADCP minus U-AVISO	0.053	0.12	0.174	0.000
U-ADCP minus U-AVISO+TG	0.051	0.11	0.188	0.000
V-ADCP minus V-AVISO	0.102	0.44	0.781	0.000
V-ADCP minus V-AVISO+TG	0.097	0.53	0.777	0.000
Mag-ADCP minus Mag-AVISO	0.070	0.25	0.492	0.069
Mag-ADCP minus Mag-AVISO+TG	0.071	0.38	0.481	0.070

**Table 3:** Standard deviations and correlations of the differences between current velocities and speeds estimated from AVISO SLA, AVISO+TG SLA fields and NH-10 ADCP (16.5 m depth) measurements as well as the slopes and y-intercepts of the linear regressions.

	**s.d. (ms^−1^)**	**R (ADCP versus AVISO(+TG))**	**Slope**	**Intercept**
U-ADCP minus U-AVISO	0.088	0.55	0.893	0.000
U-ADCP minus U-AVISO+TG	0.096	0.59	3.786	0.000
V-ADCP minus V-AVISO	0.176	0.51	1.378	0.000
V-ADCP minus V-AVISO+TG	0.142	0.73	0.819	0.000
Mag-ADCP minus Mag-AVISO	0.128	0.27	0.743	0.121
Mag-ADCP minus Mag-AVISO+TG	0.121	0.51	0.621	0.096

**Table 4:** Standard deviations and correlations of the differences between current velocities and speeds estimated from AVISO SLA, AVISO+TG SLA fields and M2 ADCP (15 m depth) measurements as well as the slopes and y-intercepts of the linear regressions.

	**s.d. (ms^−1^)**	**R (ADCP versus AVISO(+TG))**	**Slope**	**Intercept**
U-ADCP minus U-AVISO	0.111	0.07	0.222	0.000
U-ADCP minus U-AVISO+TG	0.108	0.13	0.510	0.000
V-ADCP minus V-AVISO	0.136	0.19	0.453	0.000
V-ADCP minus V-AVISO+TG	0.132	0.35	0.567	0.000
Mag-ADCP minus Mag-AVISO	0.086	0.07	0.205	0.140
Mag-ADCP minus Mag-AVISO+TG	0.092	0.11	0.186	0.138

**Table 5:** Standard deviations and correlations of the differences between current velocities and speeds estimated from AVISO SLA, AVISO+TG SLA fields and CCE-2 ADCP (17 m depth) measurements as well as the slopes and y-intercepts of the linear regressions.

	**s.d. (ms^−1^)**	**R (ADCP versus AVISO(+TG))**	**Slope**	**Intercept**
U-ADCP minus U-AVISO	0.110	0.20	0.646	0.000
U-ADCP minus U-AVISO+TG	0.128	0.29	0.318	0.000
V-ADCP minus V-AVISO	0.125	0.35	0.674	0.000
V-ADCP minus V-AVISO+TG	0.124	0.35	1.778	0.000
Mag-ADCP minus Mag-AVISO	0.093	0.06	0.111	0.143
Mag-ADCP minus Mag-AVISO+TG	0.102	0.03	0.048	0.146
