# Supplementary material for: Blended sea level anomaly fields with enhanced coastal coverage along the U.S. West Coast
Source: Sci Data. 2016 Mar 1;3:160013. doi: 10.1038/sdata.2016.13 (PMC4772653; doi:10.1038/sdata.2016.13)
Supplement: Supplementary Information [file sdata201613-s2.pdf]

Blended sea level anomaly fields with enhanced coastal coverage along the U.S. West Coast

C.M. Risien[1], P.T. Strub[1]

[1] College of Earth, Ocean, and Atmospheric Sciences, Oregon State University, Corvallis, Oregon, USA
Corresponding author: C.M. Risien (crisien@coas.oregonstate.edu)

Supplementary Information

Table of Contents

Table S1. Acoustic Doppler Current Profiler (ADCP) parameters: Locations and water depths, manufacturers, acoustic frequencies, sensor orientations, and vertical resolutions……………………………........2

Figure S1. Zonal (top) and meridional (middle) geostrophic velocities as well as geostrophic current magnitudes (bottom) estimated from AVISO (black lines) and AVISO+TG (red lines) fields at 44.625°N, 124.375°W for the period 2002 - 2004. Also shown are zonal (top) and meridional (middle) current velocities as well as current magnitudes (bottom) as measured by the NH-10 ADCP (44.64°N, 124.3°W) at 16.5 m depth (blue lines). The mean value for each zonal and meridional time series, for the period presented here, was removed……3

Figure S2. AVISO+TG (top panels) and AVISO (bottom panels) January, April, July and October sea level anomaly (colour) and geostrophic surface velocity (vectors) climatology fields. The black line indicates the 200 meter isobaths……4

Figure S3. EOF Mode 1 for AVISO+TG, 3x3 Median Filtered AVISO+TG, and 5x5 Median Filtered AVISO+TG………..5

Data Citation.………..5

Mooring	Location and Depth	Approximate Temporal Coverage	Sensor and Manufacturer	Orientation	Bin Size
RINO	47.01°N, 124.49°W 80 m	Jun - Sep 2005 & 2006	300 kHz RDI ADCP	Downward looking	0.5 m
NH-10 (‘99-‘04) NH-10 (‘06-‘12)	44.64°N, 124.3°W 81 m	1999 - 2012 (Jan 2005 - Jun 2006 missing)	500 kHz SonTek ADP 300 kHz RDI ADCP	Upward looking Downward looking	2 m 2 m
M2	36.70°N, 122.39°W 1800 m	Jun 1998 - Aug 1999 Jun - Dec 2000 Apr 2004 - Mar 2006 Apr 2007 - Apr 2009	300 kHz RDI ADCP	Downward looking	5 m
CCE-2	34.2°N, 120.7°W 770 m	Apr 2012 - Oct 2014	75 kHz RDI ADCP	Downward looking	10 m

Table S1. Acoustic Doppler Current Profiler (ADCP) parameters: Locations and water depths, manufacturers, acoustic frequencies, sensor orientations, and vertical resolutions

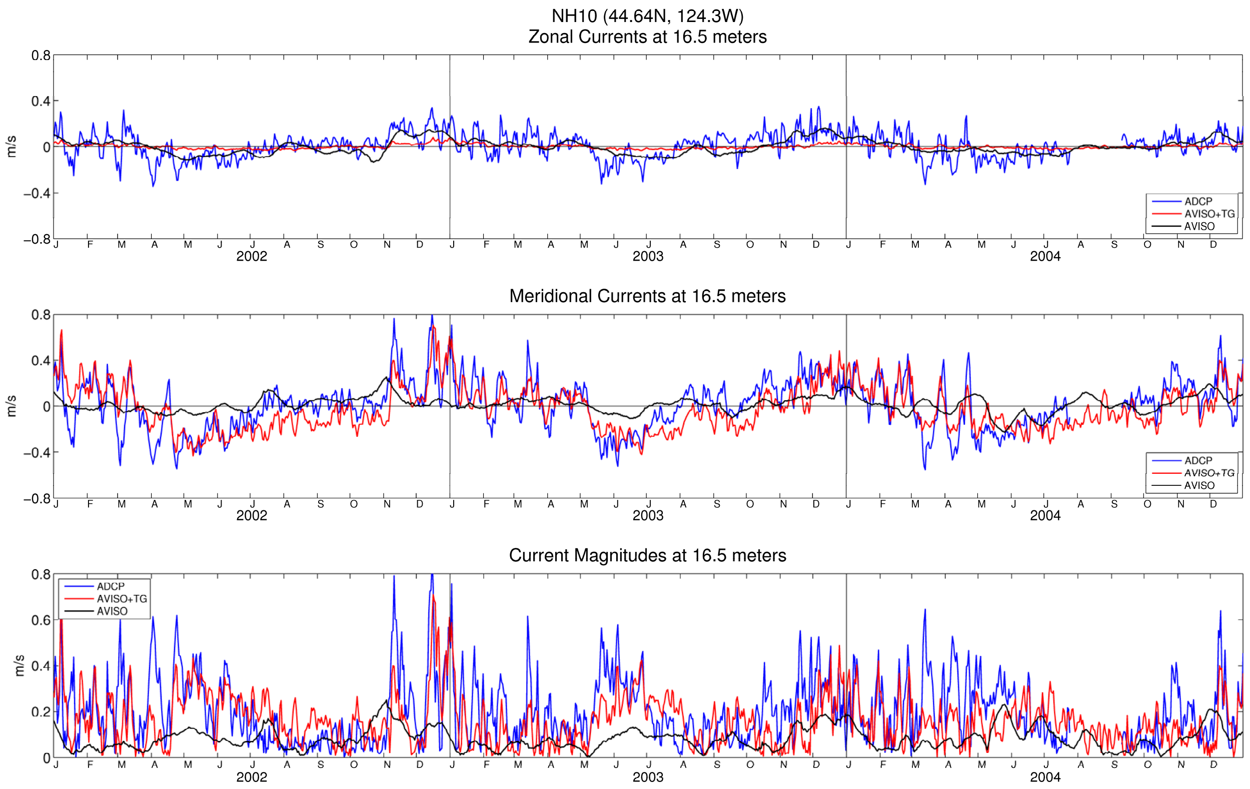

Figure S1. Zonal (top) and meridional (middle) geostrophic velocities as well as geostrophic current magnitudes (bottom) estimated from AVISO (black lines) and AVISO+TG (red lines) fields at 44.625°N, 124.375°W for the period 2002 - 2004. Also shown are zonal (top) and meridional (middle) current velocities as well as current magnitudes (bottom) as measured by the NH-10 ADCP (44.64°N, 124.3°W) at 16.5 m depth (blue lines). The mean value for each zonal and meridional time series, for the period presented here, was removed.

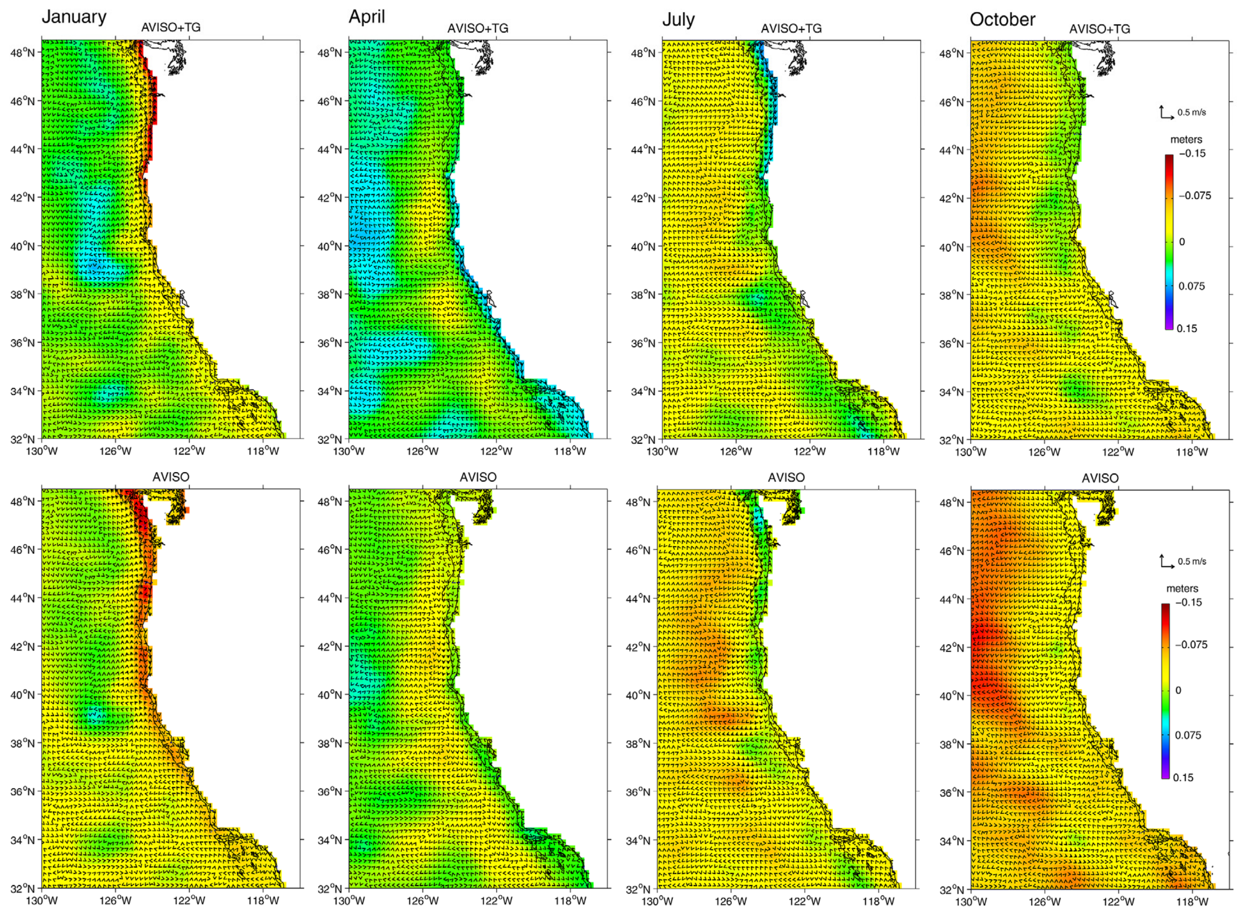

Figure S2. AVISO+TG (top panels) and AVISO (bottom panels) January, April, July and October SLA and geostrophic surface velocity (vectors) climatology fields. The black line indicates the 200 meter isobath.

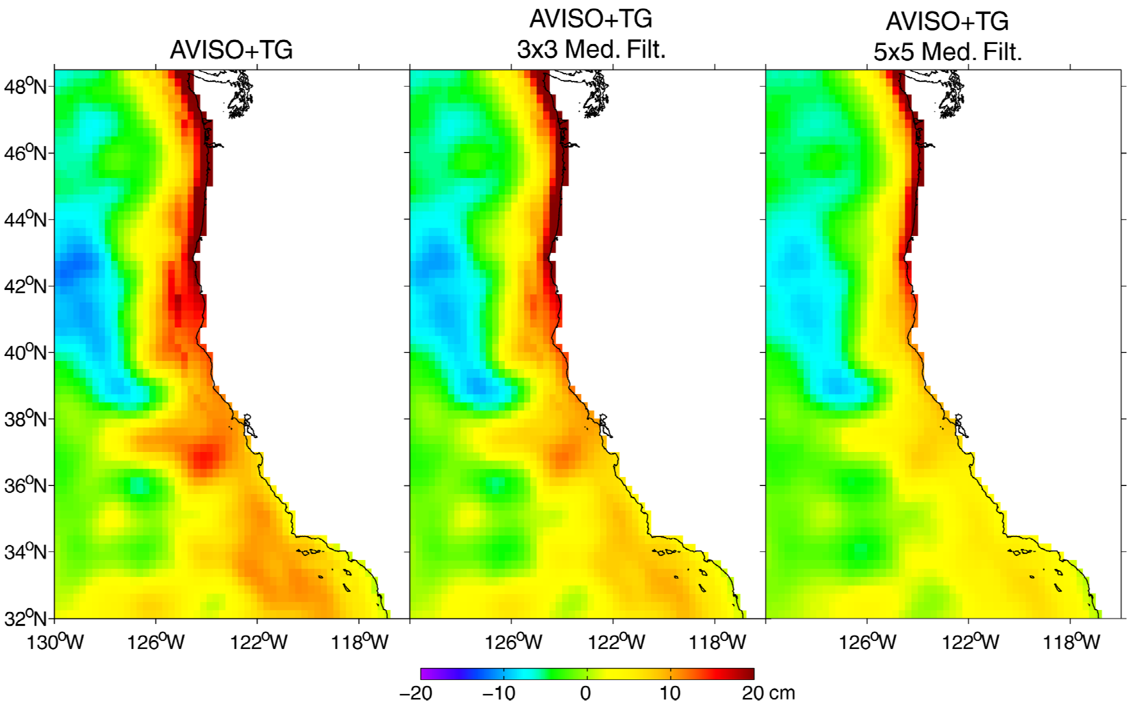

Figure S3. EOF Mode 1 for AVISO+TG, 3x3 Median Filtered AVISO+TG, and 5x5 Median Filtered AVISO+TG.

Data Citation

1. Risien, C.M. & Strub, P.T. *ScholarsArchive@OSU*. http://dx.doi.org/10.7267/N90Z7153 (2015).
